# SIRT2 in age-related neurodegenerative disorders

**DOI:** 10.18632/aging.101397

**Published:** 2018-03-03

**Authors:** Stéphane Fourcade, Tiago F. Outeiro, Aurora Pujol

**Affiliations:** 1 Neurometabolic Diseases Laboratory, Bellvitge Biomedical Research Institute (IDIBELL), Gran Via 199, L’Hospitalet de Llobregat, Barcelona, Spain; 2 CIBERER U759, Center for Biomedical Research on Rare Diseases, Valencia, Spain; 3 Institute of Neuroscience, The Medical School, Newcastle University, Framlington Place, Newcastle Upon Tyne NE2 4HH, UK.; 4 Department of Experimental Neurodegeneration, Center for Nanoscale Microscopy and Molecular Physiology of the Brain (CNMPB), Center for Biostructural Imaging of Neurodegeneration, University Medical Center Göttingen 37073 Göttingen, Germany; 5 Max Planck Institute for Experimental Medicine, Göttingen, Germany; 6 Catalan Institution of Research and Advanced Studies (ICREA), Barcelona, Spain

**Keywords:** sirtuins, SIRT2, neurodegeneration, mitochondria dysfunction

Sirtuin 2 (SIRT2) is one of seven members of the NAD^+^-dependent histone deacetylases (HDAC) family of proteins. Sirtuins play diverse roles in cellular metabolism and the aging process. SIRT2 is located in the nucleus, cytoplasm, and mitochondria, is highly expressed in the central nervous system (CNS), and has been reported to regulate a variety of processes including oxidative stress, genome integrity, and myelination [1].

Very recently, we and others found that *Sirt2*^-/-^ mice display an alteration of mitochondrial content and morphology, which results in energy failure and redox dyshomeostasis in the CNS [2,3].The axonal degeneration accompanied by redox/mitochondrial dysfunction in aged *Sirt2*^-/-^ mice [2], is also found in most of the age-related neurodegenerative disorders such as Alzheimer’s disease (AD), Parkinson’s disease (PD), or Amyotrophic Lateral Sclerosis (ALS), as well in common and rare diseases of myelin [4] and in physiological aging. This suggests that SIRT2 may play a role in the pathogenesis of these disorders, which may have strong therapeutic value. Furthermore, the effects of SIRT2 on myelination are intriguing, and have been examined *in vivo* in the peripheral nervous system, showing that both absence or overexpression are detrimental (Beirowski et al. 2011). This illustrates that tight control of Sirt2 dosage is a critical factor for successful myelin formation. The same is true concerning oligodendroglial precursor cell cultures, which show a time and dosage-dependent control of SIRT2 expression during differentiation, mediated by QKI proteins (Thangaraj et al. 2017).

In the last 10 years, it has been suggested that SIRT1 and SIRT2 play in a yin-yang relationship in which either activation of SIRT1 or inhibition of SIRT2, produced beneficial effects on neurodegenerative disorders [1]. However, in the light of recent reports indicating that inhibition of SIRT2 is either detrimental or has no positive effects on neurodegenerative disorders such as ALS or cerebral ischemia [1], the fact that pharmacological and/or genetic inhibition of SIRT2 ameliorates pathology in models of AD and PD raises a double question: What is the molecular basis for this selectivity? Why were no deleterious effects observed in the latter models?

The first answer appears straight-forward: In PD, inhibition of SIRT2 reduces α-synuclein aggregation and toxicity by modifying the levels of acetylation of this protein [5]; in AD, SIRT2 is abnormally overexpressed and deacetylates tubulin which results in microtubule destabilization, Tau dissociation from microtubules, and subsequent oligomerization and aggregate formation [6]. Thus, SIRT2 seems to act directly on the cause instead of the downstream consequences of the pathology, i.e redox homeostasis and mitochondria malfunction, in both PD and AD models in which there were no obvious deleterious effects due to Sirt2 reduction. However, we have also shown that SIRT2 is important for dopaminergic differentiation (Szego et al. 2017), and that it can suppress microglial activation and brain inflammation [1]. These findings suggest that SIRT2 may play different roles in different cell types and during different stages of development/aging. In addition, other possibilities may account for the differential effects of modulating SIRT2: i) a compensatory effect leading to increased SIRT1 expression, as shown in our study [2], a non-negligible phenomenon not explored in the AD and PD models described; or ii) a direct impact on the limited pool of NAD^+^, a main metabolic regulator linking cellular metabolism to changes in signaling and transcriptional events, through redox enzymatic reactions or through NAD^+^-consuming enzymes such as sirtuins or PARP-1, among others [7].

In conclusion, the connection between SIRT2 and neurological disorders is now well established, and thus, the potential of SIRT2 as a therapeutic target deserves to be studied in greater depth. We believe that any positive results obtained with either activators or inhibitors of SIRT2 demands cautious investigation on the mechanisms and idiosyncrasies of each particular model, for optimizing translational efforts while minimizing the risk for detrimental effects.
